# Supplementary material for: Development of a Normal Porcine Cell Line Growing in a Heme-Supplemented, Serum-Free Condition for Cultured Meat
Source: Int J Mol Sci. 2024 May 27;25(11):5824. doi: 10.3390/ijms25115824 (PMC11172042; doi:10.3390/ijms25115824)
Supplement: Supplementary file 1 [file ijms-25-05824-s001.zip › ijms-2994726-supplementary.pdf]

이 문서는 서울아산병원의 문서입니다. 동의없이 수정, 변경 및 복사할 수 없습니다.

# Supplementary Information

Supplementary Figure 1-6

Supplementary Table 1-3

서울아산병원 Asan Medical center

이 문서는 서울아산병원의 문서입니다. 동의없이 수정, 변경 및 복사할 수 없습니다.

**Figure S1**

A

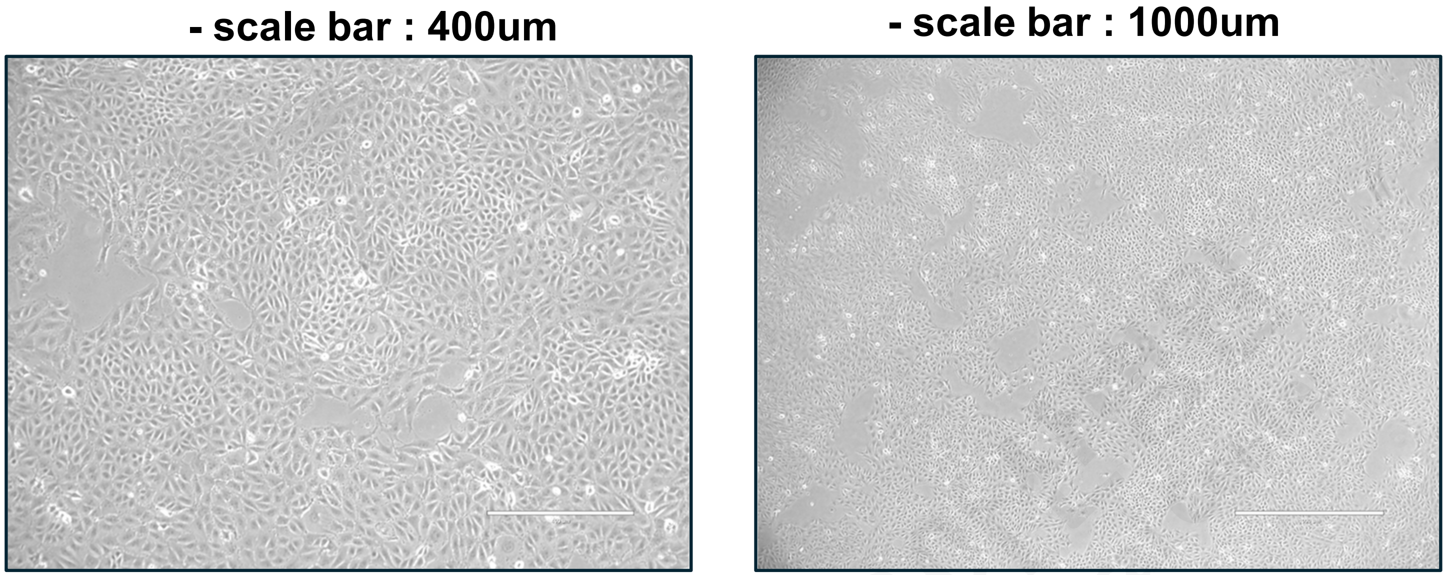


B

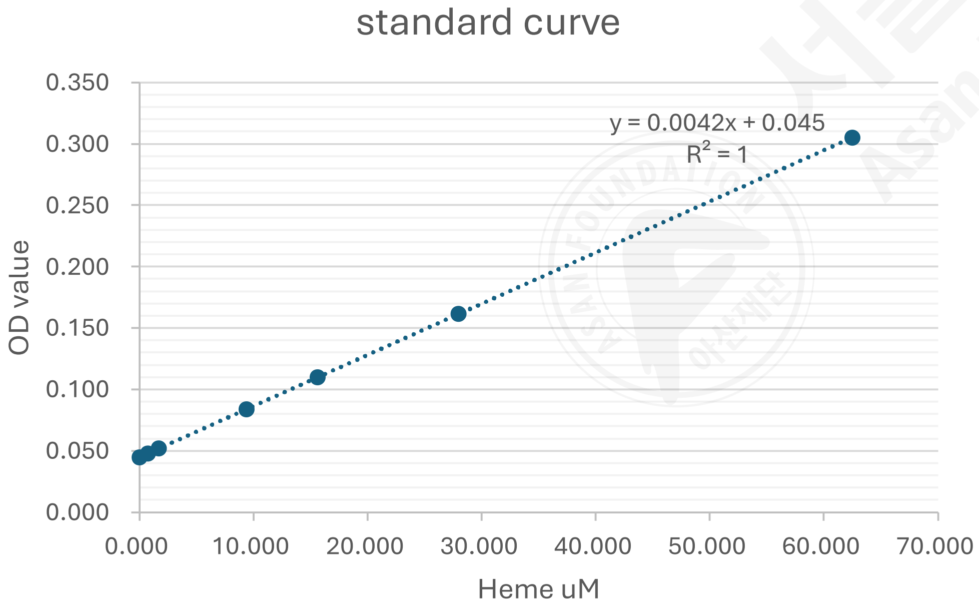


**Supplementary Figure 1.** Culture of PK15 cells and measurement of heme in bacterial extract to establish heme adaptation condition. **A.** Representative pictures of PK15 under 2% of FBS. **B.** Standard Curve for the quantitation of Heme in bacterial extract. See methods for details.

이 문서는 서울아산병원의 문서입니다. 동의없이 수정, 변경 및 복사할 수 없습니다.

# Figure S2

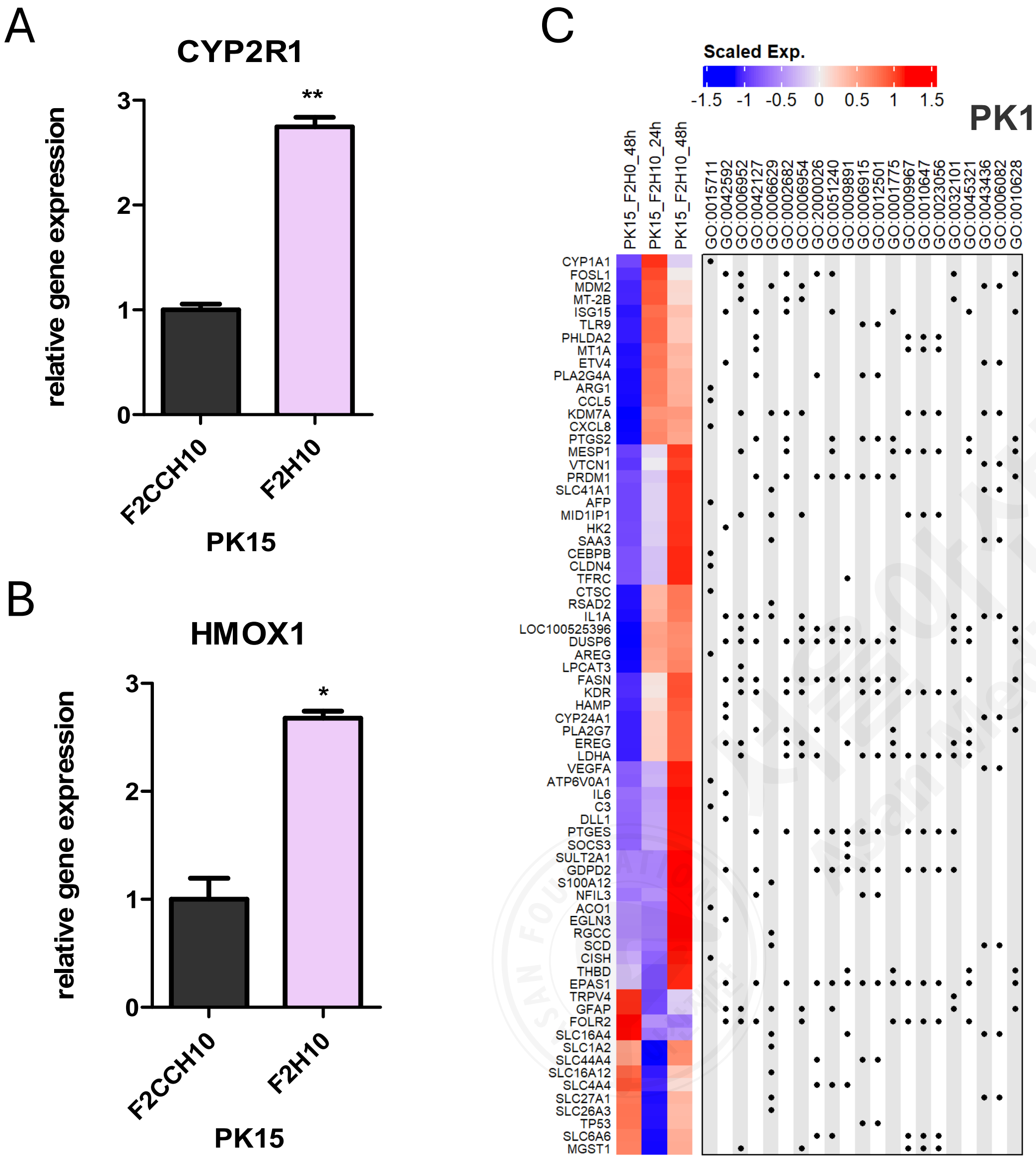


**PK15_F2H0_48h - PK15_F2H10_24h - PK15_F2H10_48h**

| ID | Description |
|---|---|
| GO:0015711 | organic anion transport |
| GO:0042592 | homeostatic process |
| GO:0006952 | defense response |
| GO:0042127 | regulation of cell population proliferation |
| GO:0006629 | lipid metabolic process |
| GO:0002682 | regulation of immune system process |
| GO:0006954 | inflammatory response |
| GO:2000026 | regulation of multicellular organismal development |
| GO:0051240 | positive regulation of multicellular organismal process |
| GO:0009891 | positive regulation of biosynthetic process |
| GO:0006915 | apoptotic process |
| GO:0012501 | programmed cell death |
| GO:0001775 | cell activation |
| GO:0009967 | positive regulation of signal transduction |
| GO:0010647 | positive regulation of cell communication |
| GO:0023056 | positive regulation of signaling |
| GO:0032101 | regulation of response to external stimulus |
| GO:0045321 | leukocyte activation |
| GO:0043436 | oxoacid metabolic process |
| GO:0006082 | organic acid metabolic process |
| GO:0010628 | positive regulation of gene expression |

**Supplementary Figure 2.** RNA-seq analysis of PK15 cells treated with Heme extract show distinct gene expression alteration. **A and B**. Validation of expression changes of CYP2R1(A) or HMOX1(B) upon the treatment of Heme extracts (marked as F2H10, pink bar). As a control, the bacterial extract without heme (marked as F2CCH10) is shown (black bar). **C.** Summary of GO analysis for the time-course treatment of Heme extracts in PK15 cells. Left panel shows heat map of key genes altered during the heme treatment time (24 and 48 hrs) presented with corresponding functions in GO analysis. The description for selected GO is shown in Table on right.

이 문서는 서울아산병원의 문서입니다. 동의없이 수정, 변경 및 복사할 수 없습니다.

# Figure S3

A

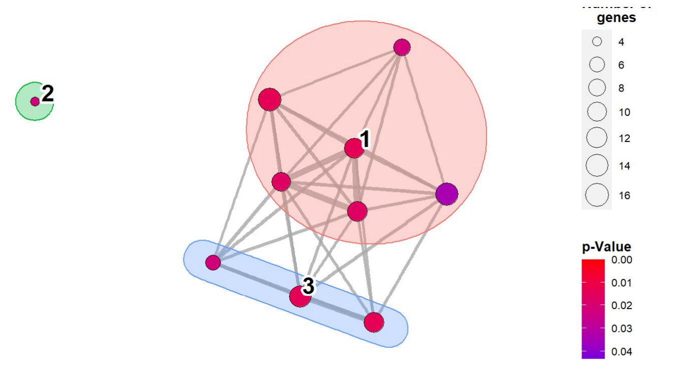


| Cluster | GO terms in Cluster |
|---|---|
| 1 | regulation of multicellular organismal development, positive regulation of protein metabolic process, blood vessel development, ... |
| 2 | circadian regulation of gene expression, ... |
| 3 | enzyme linked receptor protein signaling pathway, transmembrane receptor protein tyrosine kinase signaling pathway, protein kinase B signaling, ... |

B

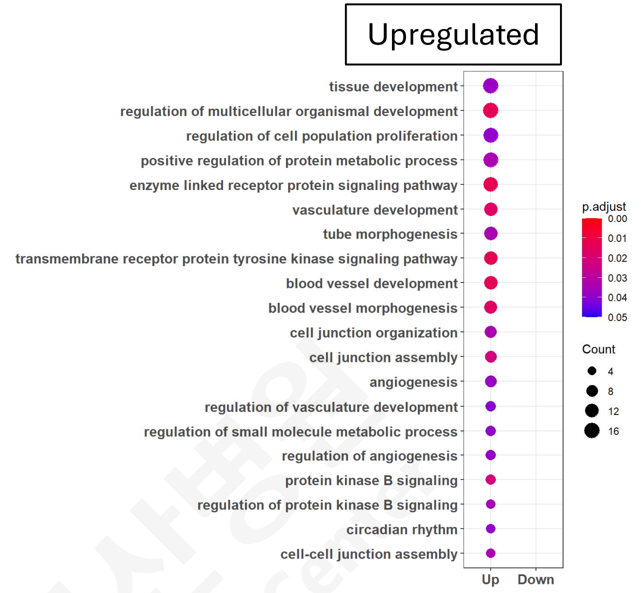


C

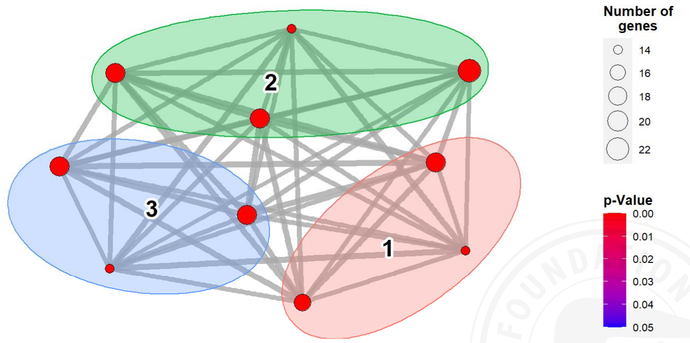


| Cluster | GO terms in Cluster |
|---|---|
| 1 | biological process involved in interspecies interaction between organisms, innate immune response, defense response to virus, ... |
| 2 | defense response, response to other organism, response to biotic stimulus, ... |
| 3 | defense response to other organism, response to external biotic stimulus, defense response to symbiont, ... |

D

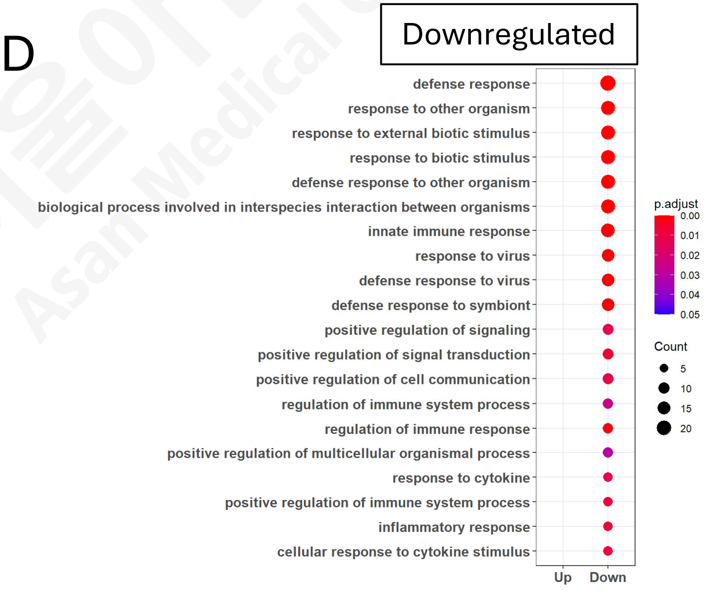


**Supplementary Figure 3.** Clustering analysis of gene expression changes under transient heme treatment in PK15 cells. **A.** Representative upregulated three clusters and its GO terms. The interaction among genes are marked as a grey lines. The size of dots indicate number of genes and color shows p-value. **B.** Upregulated biological functions enriched in the GO analysis of PK15 cells with heme treatment. The size of dots indicate the number of genes involved and color shows p-values. **C.** Representative down-regulated three clusters and its GO terms. The interaction among genes are marked as a grey lines. The size of dots indicate number of genes and color shows p-value. **D.** Down-regulated biological functions enriched in the GO analysis of PK15 cells with heme treatment. The size of dots indicate the number of genes involved and color shows p-values.

이 문서는 서울아산병원의 문서입니다. 동의없이 수정, 변경 및 복사할 수 없습니다.

**Figure S4**

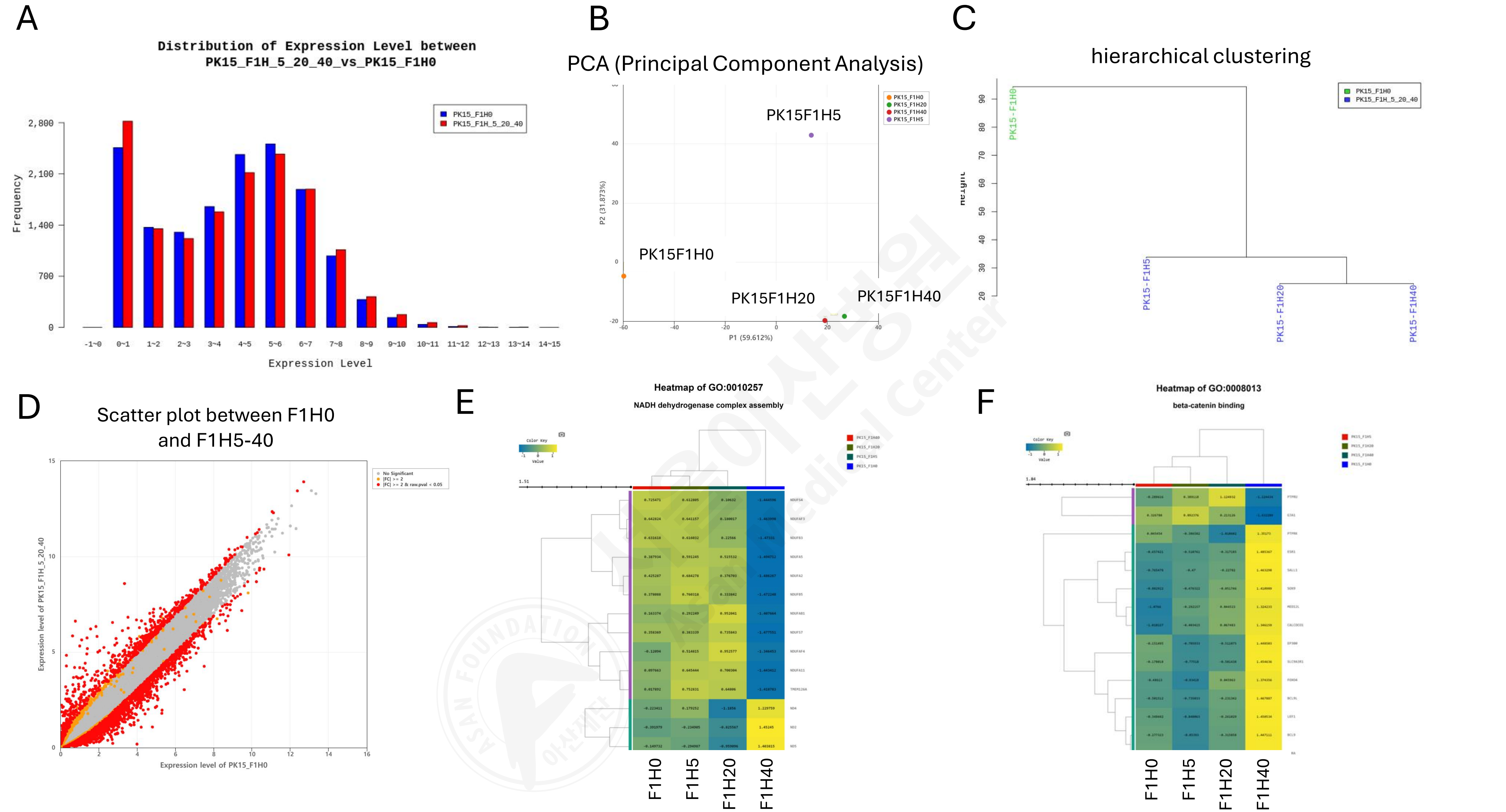


**Supplementary Figure 4.** RNA seq analysis of the heme-adapted PK15 cells (PK15H) **A.** Distribution of expression level between control (F1H10, blue bars) and Heme treated (F1H5-40, red bars) PK15H cells. **B.** PCA plot analysis of control (F1H0, Orange) and heme treated (F1H5, 20, 40 in purple, green and red dots respectively). **C.** Hierarchical clustering result of the heme treated PK15 cells. Control (F1H0, in green) and heme treated (F1H5, 20, 40, in blue) are marked. **D.** Scatter plot showing the expression correlation between control (F1H10, x axis) and Heme treated (F1H5-40, y axis) PK15H cells. Red dots are indicate significantly altered genes, **E and F.** A representative GO analysis result indicating the upregulation of NADH dehydrogenase complex assembly (E) or down-regulation of beta-catenin binding (F) in PK15H cells. The name of each gene is marked on right end and the fold change of expression is indicated by color code, with numbers in each box.

이 문서는 서울아산병원의 문서입니다. 동의없이 수정, 변경 및 복사할 수 없습니다.

**Figure S5**

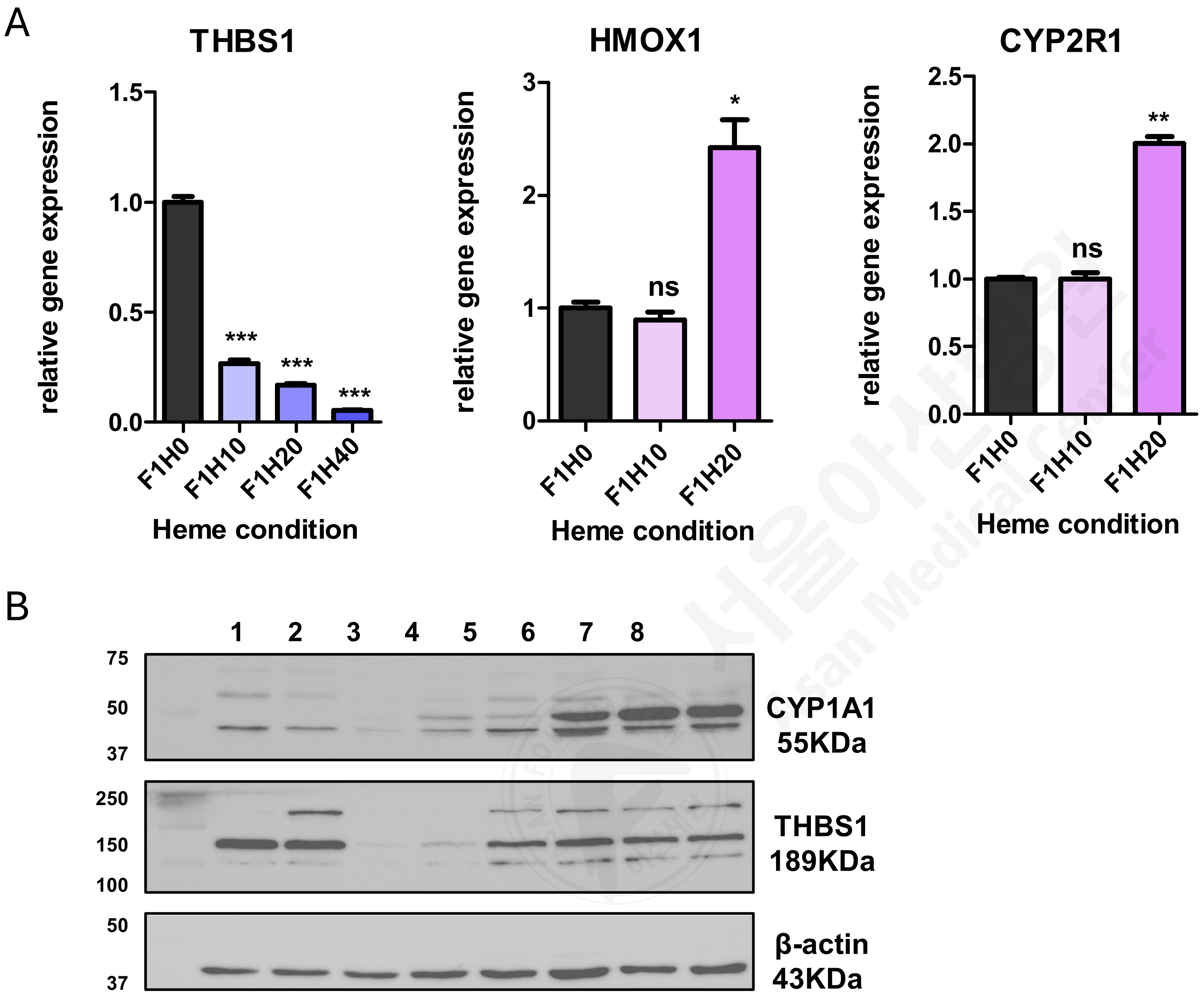


**Supplementary Figure 5.** Validation of altered gene expression in the Heme adapted PK15H cells. **A.** Real-time PCR results of THBS1, HMOX and CYP2R1 in control (F1H10, black bar) or heme adapted (F1H10, 20 40) cells. ns; not significant, * $p<0.05$, ** $p<0.005$, *** $p<0.001$. **B.** Western blot results for the CYP1A1, THBS1 and Actin. Lane information ; 1. HeLa – CYP1A1 Positive Control, 2. MDA MB 231-THBS1 Positive Control, 3. PK15 F1, 4. PK15 F1 H5, 5. PK15 F1 H20, 6. PK15 F1 H40, 7. PK15 F0 H5, 8. PK15 F0 H10.

이 문서는 서울아산병원의 문서입니다. 동의없이 수정, 변경 및 복사할 수 없습니다.

**Figure S6**

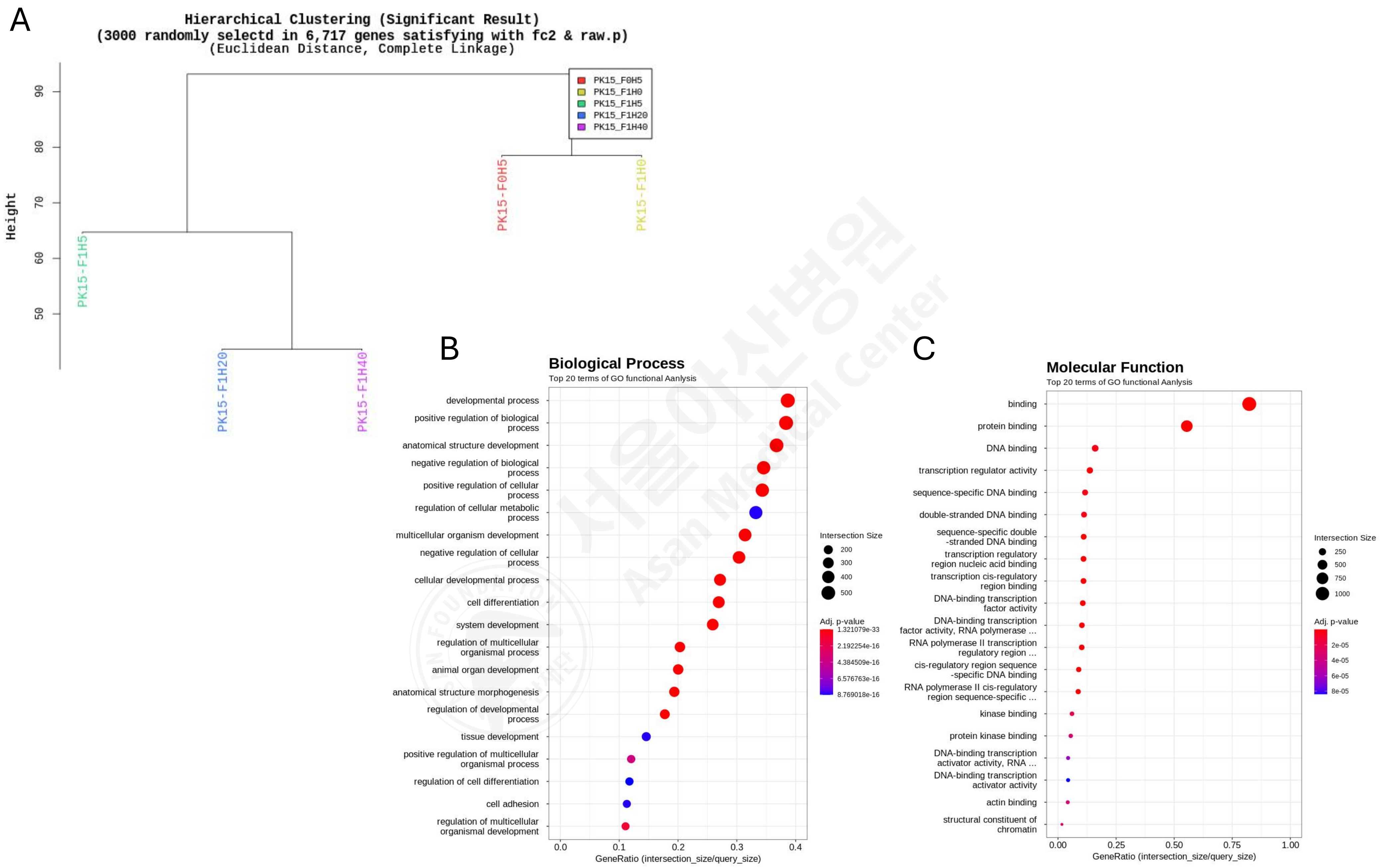


**Supplementary Figure 6.** RNA seq analysis of serum free adapted PK15H cells. **A.** Hierarchical clustering analysis of the serum free adapted PK15H cells (F0H5, in red) compared with control (F1H0, in yellow) or PK15H cells. **B and C.** A representative result of biological process (B) or Molecular function (C) that is altered during the serum free adaptation of PK15H cells. Top 20 items are listed on left of each panel and the size of dots indicate number of genes involved. The color of dots indicates p-value.

의과대학/G12**10

2024-05-15 17:27:48

이 문서는 서울아산병원의 문서입니다. 동의없이 수정, 변경 및 복사할 수 없습니다.

**Supplementary Table 1.** List of top differentially expressed genes in PK15H cells under serum free condition supplemented with heme extracts

| Transcript_ID | Gene_ Symbol | Description | PK15_F0H5/ PK15_F1H5.fc | PK15_F0H5/ PK15_F1H5.logCPM | PK15_F0H5/ PK15_F1H5.raw.pval | PK15_F0H5/ PK15_F1H5.bh.pval | PK15_F1H5. mean | PK15_F0H5. mean |
|---|---|---|---|---|---|---|---|---|
| NM_214009,XM_021080819 | C3 | complement C3 | 77.066497 | 6.171716 | 4.793E-118 | 7.241E-114 | 1.508482 | 7.163357 |
| XM_003127647,XM_021095426 | PADI1 | peptidyl arginine deiminase 1, transcript variant X2 | 283.764993 | 3.621244 | 2.24E-101 | 1.6924E-97 | 0.117042 | 4.674612 |
| NM_213774,XM_021080276,XM_021080278,XM_021080282 | CD74 | CD74 molecule, major histocompatibility complex, class II invariant chain | 94.870023 | 2.267426 | 9.9287E-65 | 5.0004E-61 | 0.135658 | 3.397643 |
| XM_021085301,XM_021085302,XM_021085303,XM_021085304,XM_021085305,XM_021085306,XM_021085307,XM_021085308,XM_021085309,XM_021085310,XM_021085311,XM_021085312,XM_021085313,XM_021085314,XM_021085315,XM_021085316 | NAV2 | neuron navigator 2, transcript variant X16 | 16.544614 | 5.561055 | 4.1524E-64 | 1.5685E-60 | 2.673643 | 6.492607 |
| NM_001114062,NM_001130224,XM_005665844 | SLA-DQA1 | MHC class II histocompatibility antigen SLA-DQA | 21.873436 | 3.653308 | 6.2962E-62 | 1.9026E-58 | 1.069435 | 4.647983 |
| XM_005670100,XM_021070120,XM_021070121,XM_021070122,XM_021070123,XM_021070124,XM_021070125,XM_021070126,XM_021070127,XM_021070128,XM_021070129,XM_021070130,XM_021070131,XM_021070132,XM_021070133,XM_021070134,XM_021070135 | PIGZ | phosphatidylinositol glycan anchor biosynthesis class Z, transcript variant X2 | 128.187241 | 1.911114 | 9.4082E-61 | 2.3691E-57 | 0.079073 | 3.082869 |
| XM_001926072,XM_013989002,XM_013989003,XM_021098858,XM_021098859,XM_021098861,XM_021098862 | ARNT2 | aryl hydrocarbon receptor nuclear translocator 2, transcript variant X5 | 16.798796 | 4.027541 | 8.4217E-58 | 1.8178E-54 | 1.501322 | 4.990506 |
| XM_021069364,XM_021069365 | PLXND1 | plexin D1, transcript variant X2 | 17.813055 | 3.495183 | 1.2097E-55 | 2.2846E-52 | 1.135670 | 4.482788 |
| NM_001267694,XM_013991587,XM_021068574 | CP | ceruloplasmin | 33.858171 | 2.230803 | 1.0517E-53 | 1.589E-50 | 0.342112 | 3.339944 |
| XM_013994444,XM_021083199,XM_021083200 | KIAA1549L | KIAA1549 like, transcript variant X1 | 27.684400 | 2.453641 | 1.0174E-53 | 1.589E-50 | 0.464970 | 3.533655 |
| XM_021094021,XM_021094022,XM_021094023 | CDH16 | cadherin 16, transcript variant X3 | 64.682232 | 1.721597 | 4.4556E-52 | 6.1199E-49 | 0.135658 | 2.907454 |
| XR_001301430 | LOC106506243 | uncharacterized LOC106506243 | 10.289950 | 5.180499 | 1.4588E-47 | 1.8368E-44 | 2.891935 | 6.068394 |
| XM_003129745,XM_003357234,XM_013979248,XM_013979249,XM_021062621,XM_021062622,XM_021062623 | NOX4 | NADPH oxidase 4, transcript variant X4 | -18.220396 | 2.654489 | 4.7169E-46 | 5.4822E-43 | 3.680000 | 0.719578 |
| XM_001925047 | SERPINB7 | serpin family B member 7 | -16.677017 | 2.684923 | 5.3206E-45 | 5.7421E-42 | 3.702027 | 0.781197 |
| gene-ND5 | ND5 | NADH dehydrogenase subunit 5 | 8.475249 | 7.662935 | 7.2039E-44 | 7.2562E-41 | 5.452050 | 8.505999 |
| NM_214277,XM_005653523,XM_005653524,XM_021098304 | ANPEP | alanyl aminopeptidase, membrane | 31.361824 | 1.580816 | 1.6669E-43 | 1.5741E-40 | 0.242574 | 2.766031 |
| XM_021091898 | TSFM | Ts translation elongation factor, mitochondrial | 9.938587 | 3.818296 | 3.6015E-42 | 3.2009E-39 | 1.838957 | 4.735223 |
| NM_001044548,XM_021066256,XM_021066257,XM_021066258 | NTN1 | netrin-1 | 8.679659 | 5.128408 | 4.6379E-42 | 3.893E-39 | 3.040101 | 5.993899 |
| NM_001244150,XM_005667370,XM_005667371 | TAGLN | transgelin | 10.104557 | 3.600623 | 1.0893E-41 | 8.6621E-39 | 1.670450 | 4.528423 |
| XM_005671181 | FAM25A | family with sequence similarity 25 member A | -8.931149 | 4.625820 | 2.5262E-41 | 1.9084E-38 | 5.501331 | 2.574837 |

의과대학/G12**10

2024-05-15 17:27:48

이 문서는 서울아산병원의 문서입니다. 동의없이 수정, 변경 및 복사할 수 없습니다.

**Supplementary Table 2.** List of top enriched pathways in PK15H cells under serum free condition supplemented with heme extracts

| term_id | term_name | adjusted_p_value | term_size | query_size | intersection_size | effective_domain_size | intersection_size_UP | intersection_size_DOWN |
|---|---|---|---|---|---|---|---|---|
| GO:0007275 | multicellular organism development | **1.3211E-33** | 3393 | 1363 | 428 | 19136 | 361 | 67 |
| GO:0048856 | anatomical structure development | **2.2573E-30** | 4360 | 1363 | 501 | 19136 | 417 | 84 |
| GO:0032502 | developmental process | **7.248E-30** | 4699 | 1363 | 527 | 19136 | 435 | 92 |
| GO:0048731 | system development | **9.7825E-26** | 2827 | 1363 | 353 | 19136 | 302 | 51 |
| GO:0048518 | positive regulation of biological process | **1.7998E-23** | 4924 | 1363 | 523 | 19136 | 428 | 95 |
| GO:0048523 | negative regulation of cellular process | **2.2981E-23** | 3617 | 1363 | 414 | 19136 | 337 | 77 |
| GO:0048869 | cellular developmental process | **2.2981E-23** | 3109 | 1363 | 370 | 19136 | 313 | 57 |
| GO:0030154 | cell differentiation | **3.5942E-23** | 3084 | 1363 | 367 | 19136 | 311 | 56 |
| GO:0005515 | protein binding | **9.3091E-23** | 7415 | 1313 | 728 | 17833 | 563 | 165 |
| GO:0048522 | positive regulation of cellular process | **2.0033E-22** | 4306 | 1363 | 468 | 19136 | 386 | 82 |
| GO:0048519 | negative regulation of biological process | **1.5841E-21** | 4384 | 1363 | 471 | 19136 | 372 | 99 |
| GO:0051239 | regulation of multicellular organismal process | **2.2398E-21** | 2152 | 1363 | 277 | 19136 | 228 | 49 |
| GO:0050793 | regulation of developmental process | **6.3606E-21** | 1796 | 1363 | 242 | 19136 | 206 | 36 |
| GO:0009653 | anatomical structure morphogenesis | **7.7755E-20** | 2064 | 1363 | 264 | 19136 | 235 | 29 |
| GO:0048513 | animal organ development | **1.4796E-18** | 2206 | 1363 | 273 | 19136 | 230 | 43 |
| GO:2000026 | regulation of multicellular organismal development | **1.1846E-16** | 998 | 1363 | 151 | 19136 | 128 | 23 |
| GO:0051240 | positive regulation of multicellular organismal process | **3.9689E-16** | 1139 | 1363 | 164 | 19136 | 136 | 28 |
| GO:0031323 | regulation of cellular metabolic process | **8.6713E-16** | 4452 | 1363 | 453 | 19136 | 367 | 86 |
| GO:0007155 | cell adhesion | **8.6713E-16** | 1051 | 1363 | 154 | 19136 | 136 | 18 |
| GO:0009888 | tissue development | **8.6713E-16** | 1508 | 1363 | 199 | 19136 | 178 | 21 |

의과대학/G12**10

2024-05-15 17:27:48

이 문서는 서울아산병원의 문서입니다. 동의없이 수정, 변경 및 복사할 수 없습니다.

**Supplementary Table 3**. List of primers used in this study

| Target Gene | Sense | Sequence (5’to 3’) | Annealing temperature (℃) |
|---|---|---|---|
| CYP1A1 | Forward | CCATCCCTCACAGTACCACA | 60 |
| | Reverse | TTCACCCAGTGCCTTGTTGA | |
| RGCC | Forward | GCCACTTCCACTACGAGGAG | 55 |
| | Reverse | CGAGTTTGGCTTTCCGAGGA | |
| MESP1 | Forward | AGTTGACGTGACGGTGGAAT | 60 |
| | Reverse | CCTGCTTGCCTCAAAGTGTT | |
| THBS1 | Forward | CTCAGGCCTTTCTGTGAAGG | 60 |
| | Reverse | CACCACACGAATGAAACCTG | |
| S100A10 | Forward | GCCGTCTCAAATGGAACATG | 60 |
| | Reverse | CACTGGTCCAGGTCCTTCAT | |
| ATP6V0A1 | Forward | TAGCACTGGGCACAGCATAC | 65 |
| | Reverse | GGAAAGGTCTCCAGGTCTGC | |
| PCSK1N | Forward | AAACGTCTAGAGAACCCCGC | 63 |
| | Reverse | TATTGTAGGGTCCGGAGTGG | |
| MAFF | Forward | TCTGGTGAGATAGGAGGTTG | 60 |
| | Reverse | CACAGATGGGGCTGTATCCT | |
| GPX5 | Forward | CTGTCCTCACCCTTCTGAGC | 65 |
| | Reverse | GAAGCAGGATGGCTTCTGTC | |
| CYP2R1 | Forward | GTATCCATGAGGACGCAGGT | 60 |
| | Reverse | GCCAGGAGTTGAATCAGAGC | |
| LPO | Forward | CCACCTTGACCTCTCAGCTC | 65 |
| | Reverse | GCCTCAGCCAAGACTTGTTC | |
| HMOX1 | Forward | CTTGCCCTCTGCATTCTTTC | 60 |
| | Reverse | AGCTGGACAGAGATGCTGGT | |

의과대학/G12**10

2024-05-15 17:27:48
